# Risk Factors for Poor Neurological Outcomes at Discharge in Tuberculous Meningitis: Disease Severity, Host Factors, and Drug Therapy

**DOI:** 10.3390/tropicalmed11090259

**Published:** 2026-09-14

**Authors:** Yuxia Yu, Huarui Liu, Huan Yang, Dan Ye, Lu Xia, Xiaoqing Ma, Jiayi Wang, Yuchong Sun, Feng Li, Yuanyuan Chen

**Affiliations:** 1 The Second Clinical Medical College, Zhejiang Chinese Medical University, Hangzhou 310000, China; yuyuxia_3579@163.com (Y.Y.);; 2 Center for Tuberculosis Research, Shanghai Public Health Clinical Center, Fudan University, Shanghai 200000, China; 3 Department of Tuberculosis, Hangzhou Red Cross Hospital, Hangzhou 310000, China

**Keywords:** tuberculous meningitis, neurological outcome, risk factors

## Abstract

Introduction: This study investigated independent factors associated with neurological prognosis at discharge in patients with tuberculous meningitis (TBM) and evaluated the impact of intensified treatment regimens. Methods: A retrospective analysis was conducted in 518 TBM patients from two Chinese medical centers. LASSO and multivariable logistic regression were used to identify independent risk factors for poor neurological outcomes. A 1:1 PSM based on intensified treatment exposure was performed to assess its association with neurological outcomes, with an exploratory outcome-based matched comparison conducted for other prognostic factors. Results: Among 518 patients, 123 (23.75%) had poor neurological outcomes. The poor-prognosis group had higher proportions of MRC stage III (30.08% vs. 8.35%, *p* < 0.001), linezolid use (58.54% vs. 40.76%, |SMD| = 0.361), and fluoroquinolone use (87.8% vs. 75.19%, |SMD| = 0.386). Multivariable analysis identified decreased muscle strength, MRC stage II/III, albumin < 30 g/L, and hydrocephalus as independent risk factors for poor neurological outcomes. After treatment-exposure-based PSM, intensified treatment was not significantly associated with neurological outcomes, while an exploratory matched analysis suggested a potential association between diabetes mellitus and unfavorable neurological outcomes. Conclusion: Neurological prognosis in TBM is influenced by both disease severity and host baseline status. Intensified therapy was not significantly associated with neurological outcome at discharge after adjustment, highlighting the importance of individualized risk management.

## 1. Introduction

Tuberculous meningitis (TBM) remains the most lethal and debilitating manifestation of extrapulmonary tuberculosis (TB), characterized by a profound inflammatory response following the infection of the meninges by Mycobacterium tuberculosis [1,2]. Despite global efforts, the burden of TB persists; the 2025 World Health Organization Global Tuberculosis Report indicates that while case numbers showed a slight decline in 2024, approximately 10.7 million people were affected and 1.23 million died from the disease [3]. TBM is disproportionately responsible for this mortality, with global mortality rates averaging 25% and reaching as high as 70% in high-risk regions or among people living with HIV [4]. Furthermore, over 50% of survivors suffer from permanent neurological impairments, including cognitive deficits, limb paralysis, and seizures, imposing a significant socio-economic burden on healthcare systems and families [5].

Current clinical management of TBM prioritizes early diagnosis and standardized treatment to mitigate these outcomes [6]. Prognostic assessment traditionally relies on disease severity markers, most notably the modified Medical Research Council (MRC) staging system [7]. Extensive literature has identified advanced age, late-stage disease, hydrocephalus, and impaired consciousness as key predictors of poor outcomes [8,9,10]. However, a critical limitation in existing research is that many identified prognostic factors are heavily collinear with disease severity, potentially obscuring the independent contribution of individual patient characteristics. While factors such as malnutrition (assessed via the CONUT score), low hemoglobin, and immune status are known to influence TB progression, their specific impact on TBM-related neurological functional outcomes, independent of the initial clinical stage, remains insufficiently characterized [11,12,13].

Similarly, the role of intensified treatment regimens in improving neurological outcomes is a subject of ongoing debate. While the standard intensive-phase treatment involves a four-drug regimen, several studies have explored the efficacy of adjunctive second-line agents like linezolid and fluoroquinolones [14,15]. Although these agents have shown potential in reducing mortality for drug-resistant TBM, their ability to significantly reduce overall mortality or improve neurological functional recovery in the general TBM population has not been definitively established [15]. Consequently, there is a clear need to distinguish whether improved outcomes are a product of the therapeutic regimen itself or the baseline clinical status of the patient.

This study systematically evaluated the associations of disease severity, baseline patient characteristic, and treatment regimens with neurological outcomes at discharge in a retrospective cohort of 518 patients with TBM. By applying propensity score matching (PSM) based on intensified treatment exposure, this study further assessed the association between intensified treatment and neurological outcomes, while an exploratory outcome-based matched comparison was conducted to examine other potential prognostic factors. The research objective was to facilitate early identification of high-risk patients, optimize treatment strategies and patient management, and thereby improve clinical outcomes and quality of life among patients with TBM.

## 2. Methods

### 2.1. Study Design and Population

This was a multicenter retrospective cohort analysis of patients meeting the diagnostic criteria for TBM. The study population comprised patients admitted to two specialized tuberculosis centers in China: Hangzhou Red Cross Hospital and Shanghai Public Health Clinical Center, between November 2012 and November 2023.

The inclusion criteria were: (1) age ≥ 15 years; (2) a diagnosis of TBM according to the standardized diagnostic framework, which classifies cases as confirmed (microbiological evidence), highly suspected (clinical and imaging criteria), or suspected (clinical presentation without a more plausible alternative) [1]; and (3) availability of essential laboratory and imaging data required for diagnosis and analysis.

Exclusion criteria included pregnancy or lactation, a history of major central nervous system disorders (e.g., sequelae of stroke or previous cranial surgery), missing key diagnostic or analytical variables.

This research adhered to the Declaration of Helsinki and was approved by the Ethics Committee of Hangzhou Red Cross Hospital (Approval No.: 2025-S198-001), which waived the requirement for informed consent due to the retrospective nature of this study. Hangzhou Red Cross Hospital served as the leading institution, and Shanghai Public Health Clinical Center formally recognized and relied upon this ethical approval in accordance with its institutional requirements.

### 2.2. Data Collection and Severity Assessment

Demographic characteristics, clinical symptoms, laboratory test results (blood and cerebrospinal fluid), and neuroimaging findings were extracted from electronic medical records at the time of admission.

Disease severity was staged according to the MRC system [7]: Stage I: Glasgow Coma Scale (GCS) score of 15; fully conscious with no neurological deficits; Stage II: GCS score of 11–14; presence of lethargy or cranial nerve palsies; Stage III: GCS score ≤ 10; coma or severe neurological deficits.

### 2.3. Therapeutic Intervention

During the study period (2012–2023), patients were treated according to the Chinese guidelines for tuberculosis management available at the time, including the Guidelines for Tuberculosis Control and Prevention in China (2015 edition) and subsequent updates. All patients received anti-tuberculosis therapy based on national guidelines. The standard regimen consisted of an intensive phase (2 months) of isoniazid, rifampicin, pyrazinamide, and ethambutol, followed by a continuation phase (10 months) of isoniazid, rifampicin and ethambutol. Intensified treatment was defined as the addition of linezolid, fluoroquinolones, or amikacin for at least 2 weeks. The decision to initiate intensified therapy was made by treating physicians based on individual clinical conditions, including disease severity, suspected drug resistance, and response to initial anti-tuberculosis therapy. The dosages of intensified agents were prescribed according to the approved drug labels and prescribing information, with adjustments made when clinically indicated. Once the intensified regimen was determined, it was generally maintained throughout the subsequent treatment course unless clinical conditions, adverse events, or other treatment-related considerations required adjustment. Concomitant treatments included corticosteroids to mitigate neuroinflammation, mannitol for intracranial pressure management, and supportive care for nutrition and electrolyte balance.

### 2.4. Outcome Measures

The primary outcome was the neurological status at discharge, evaluated using the modified Rankin Scale (mRS). The mRS scores range from 0 (no symptoms) to 6 (death) [16]. Patients were stratified into two cohorts: (1) favorable outcome: mRS scores 0–2; (2) unfavorable outcome: mRS scores 3–6. Given the retrospective design and the long study period (2012–2023), follow-up completeness varied substantially among patients. Therefore, only discharge neurological status was selected as the primary endpoint to avoid potential selection bias caused by incomplete long-term follow-up.

### 2.5. Statistical Analysis

Missing data were assessed before statistical analysis. For continuous variables with less than 10% missing data, missing values were imputed using the median value. Variables with substantial missingness or missing essential diagnostic information were not imputed, and the corresponding patients were excluded during the screening process. Statistical analyses were conducted using IBM SPSS Statistics version 25.0 and R software version 4.5.1. Continuous variables were expressed as the mean ± standard deviation or median (interquartile range) according to their distribution, while categorical variables were expressed as frequencies and percentages. Baseline differences between groups were evaluated using standardized mean differences (SMDs) for continuous and binary categorical variables, whereas *p* values were used for categorical variables with three or more categories.

LASSO was first applied to the entire cohort to reduce multicollinearity and identify candidate factors associated with poor neurological prognosis. Next, the selected variables were entered into a multivariable logistic regression analysis to evaluate factors independently associated with unfavorable neurological outcomes in TBM. The strength of the associations between variables was quantified using odds ratios (ORs) and their 95% confidence intervals (CIs).

To evaluate the association between intensified treatment and neurological outcomes, patients were categorized according to intensified treatment exposure. In addition, 1:1 propensity score matching (PSM) was performed based on baseline characteristics associated with treatment allocation to reduce potential confounding by indication. The logistic regression model was used to estimate the propensity scores, followed by 1:1 nearest-neighbor matching. In the treatment-exposure-matched cohort, logistic regression was used to evaluate the association between individual intensified treatment drugs and neurological outcomes. Furthermore, an exploratory matched comparison was performed using neurological outcome as the grouping variable to explore the associations of intensified treatment drugs and other clinical factors with neurological outcomes. Additionally, a sensitivity analysis excluding patients classified as suspected TBM was performed to assess the robustness of the findings with respect to diagnostic uncertainty. A *p* value < 0.05 was considered statistically significant, and all tests were two-sided.

## 3. Results

### 3.1. Patient Characteristics

**Figure 1:**
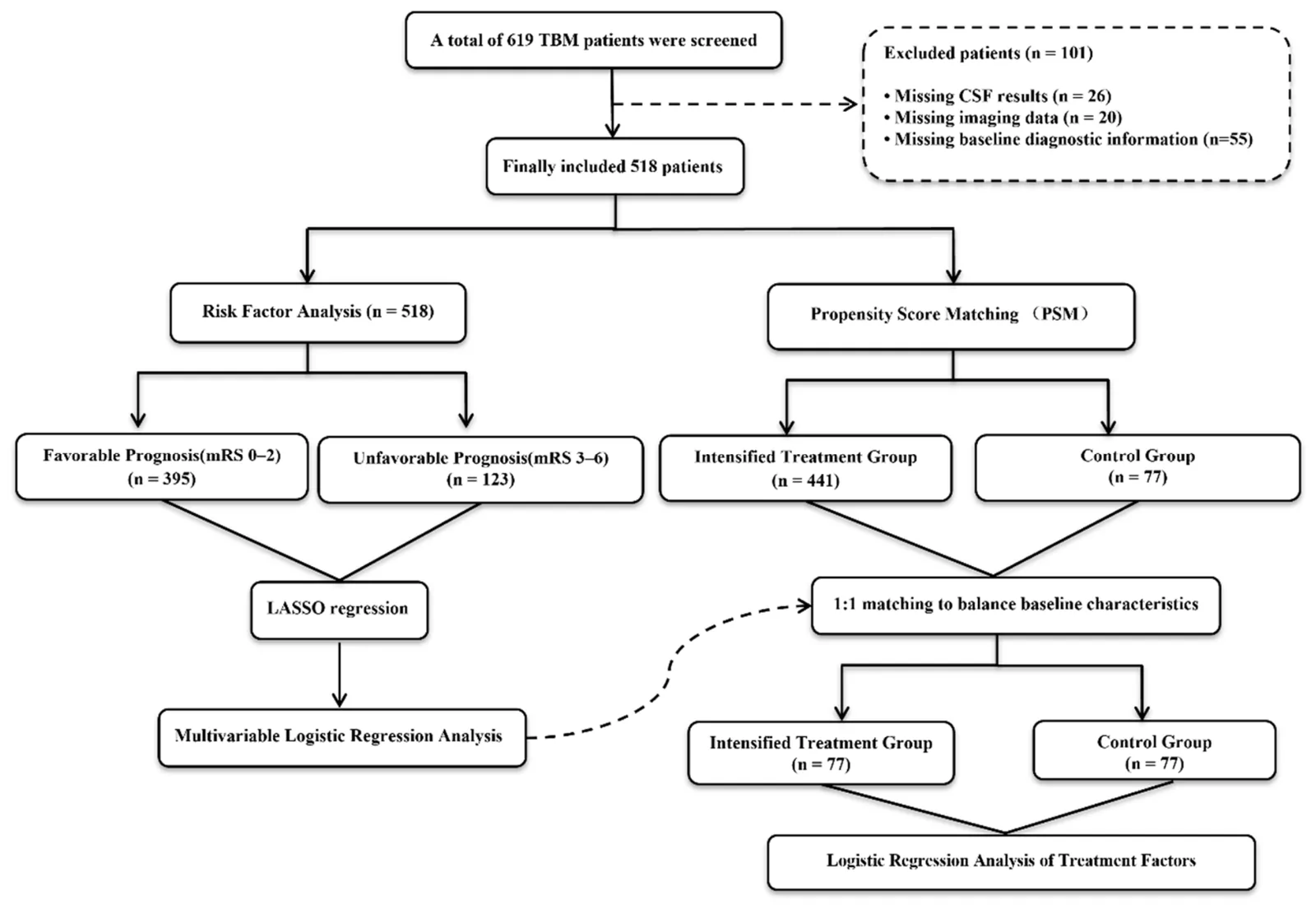
Flow diagram of patient selection and inclusion in this study.

A total of 619 patients were screened during the study period. After excluding patients with incomplete cerebrospinal fluid examinations, insufficient imaging data, missing essential baseline information, and other exclusion criteria, 518 patients were included in the final analysis. The detailed patient selection process is shown in Figure 1. Based on neurological outcomes at discharge, 395 patients (76.25%) were assigned to the favorable-prognosis group (mRS 0–2) and 123 patients (23.75%) to the unfavorable-prognosis group (mRS 3–6). Microbiological confirmation (definite TBM) via cerebrospinal fluid (CSF) culture or nucleic acid amplification testing (NAAT) was achieved in 311 cases (60.04%), while the remaining 207 (39.96%) were classified as probable or suspected TBM (Table 1).

### 3.2. Baseline Disease Severity and Clinical Manifestations

Patients with unfavorable outcomes exhibited greater baseline disease severity (Table 1). Specifically, the distribution of MRC disease stages was imbalanced between the two groups, with a higher proportion of patients classified as MRC stage III in the unfavorable outcome group (30.08% vs. 8.35%, *p* < 0.001) and a lower proportion classified as stage I (9.76% vs. 40.51%, *p* < 0.001). This finding indicated a substantial imbalance in disease severity between the two groups. Additionally, patients with unfavorable prognosis had lower GCS scores at admission (|SMD| = 0.499), which further reflected their overall unfavorable neurological functional status.

Clinical manifestations associated with unfavorable prognosis included consciousness disorder (|SMD| = 0.521), cranial nerve palsy (|SMD| = 0.217), decreased muscle strength (|SMD| = 0.509), and abnormal muscle tone (|SMD| = 0.259). Furthermore, the proportion of patients with unfavorable prognosis who were found to have hydrocephalus on imaging examinations at admission was also higher (|SMD| = 0.343).

### 3.3. Individual Factors and Laboratory Tests

Neurological outcomes differed between the two treating institutions, with a higher proportion of adverse outcomes observed among patients treated at the public hospital (|SMD| = 0.399). Compared with patients with favorable outcomes, those with poor prognosis were older (|SMD| = 0.204) and exhibited a more pronounced systemic inflammatory response, as reflected by elevated peripheral white blood cell counts (|SMD| = 0.314) and higher neutrophil-to-lymphocyte ratio (NLR) levels (|SMD| = 0.261). In addition, cerebrospinal fluid lactate dehydrogenase levels were markedly increased in the poor-prognosis group (|SMD| = 0.285), suggesting more severe central nervous system inflammation and tissue injury. Furthermore, patients with adverse outcomes demonstrated poorer nutritional and metabolic status, characterized by lower serum albumin levels (|SMD| = 0.349) and electrolyte disturbances, including elevated serum potassium levels (|SMD| = 0.287) (Table 2).

### 3.4. Therapeutic Interventions and Intensified Regimens

Analysis of treatment regimens revealed that intensified-treatment agents were more frequently administered to patients who ultimately experienced poor outcomes. The use of linezolid (58.54% vs. 40.76%, |SMD| = 0.361) and fluoroquinolones (87.80% vs. 75.19%, |SMD| = 0.386) was higher in the unfavorable outcome group, indicating an imbalance in treatment exposure between the two groups (Table 3). Several adverse events occurred numerically more frequently in the intensified treatment group; however, only rash differed significantly between groups (Supplementary Table S1).

### 3.5. Feature Selection via LASSO Regression and Multivariable Analysis

**Figure 2:**
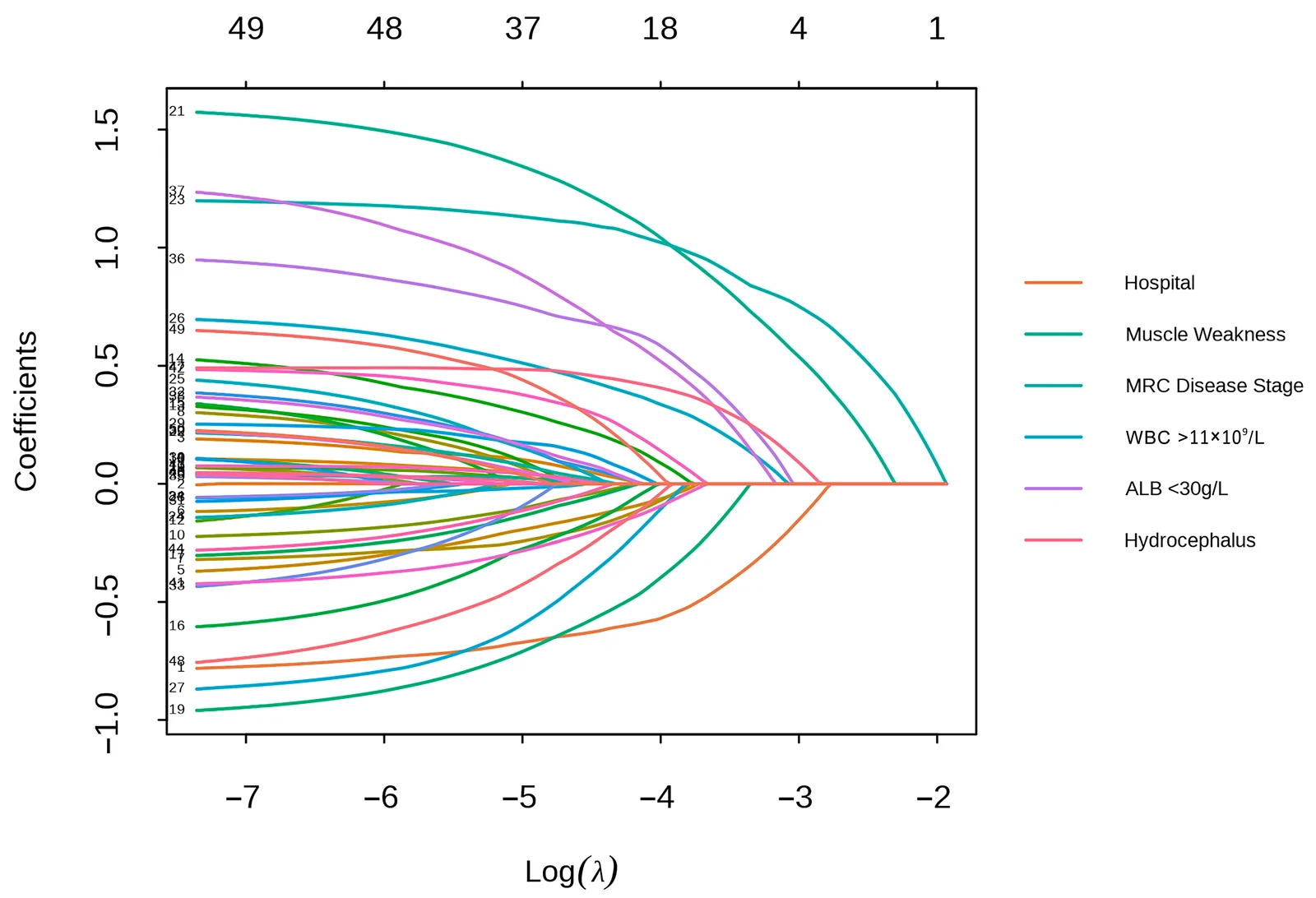
Path Diagram of Characteristic Coefficients for Variable Selection in LASSO Regression. *Figure Legend*: This figure shows the variation paths of regression coefficients of each candidate variable with the regularization parameter λ in LASSO (Least Absolute Shrinkage and Selection Operator) regression analysis. As λ increases, the regression coefficients of unimportant variables are gradually shrunk to 0, which is used to screen the most relevant predictors for the neurological prognosis of TBM patients.

**Figure 3:**
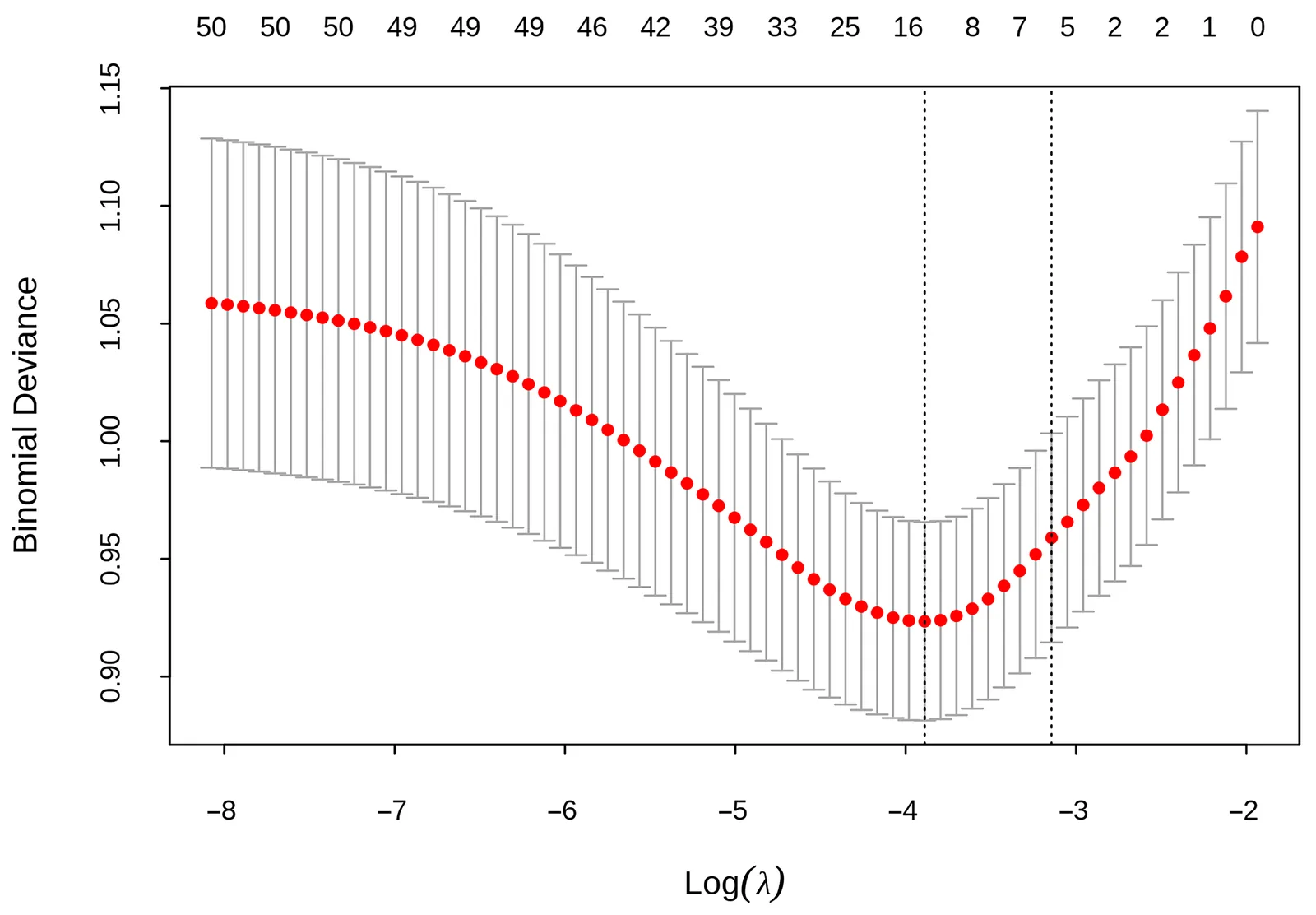
10-Fold Cross-Validation Results and Optimal λ Value Selection Diagram of LASSO Regression. *Figure Legend*: This figure displays the error variation in the LASSO regression model based on 10-fold cross-validation. Red dots represent the mean binomial deviance values, and error bars indicate the standard errors. λ.min represents the λ value that minimizes the cross-validation error; λ.1se represents the maximum λ value within one standard error of the minimum error.

LASSO regression identified candidate variables associated with unfavorable neurological outcomes at discharge. Based on the lambda.1SE criterion, the optimal penalty parameter (λ) was 0.04. The variables selected by LASSO included hospital, muscle strength decline, MRC disease stage, white blood cell count (WBC), albumin (ALB), and hydrocephalus (Figure 2 and Figure 3).

Although hospital was identified as a potential factor during variable selection, it was considered a center-level characteristic rather than an individual biological predictor. Therefore, the primary multivariable analysis excluded hospital to focus on patient-level clinical factors. A sensitivity analysis including hospital as an adjustment variable was additionally performed and is presented in Supplementary Table S2. Given its clinical relevance, age was additionally included in the multivariable model.

In the primary multivariable analysis without hospital, decreased muscle strength (OR 3.63, 95% CI 2.12–6.21; *p* < 0.001), MRC stage II (OR 2.56, 95% CI 1.26–5.21; *p* = 0.009), MRC stage III (OR 10.18, 95% CI 4.62–22.42; *p* < 0.001), white blood cell count > 11 × 10^9^/L (OR 2.13, 95% CI 1.13–4.00; *p* = 0.019), serum albumin < 30 g/L (OR 2.39, 95% CI 1.29–4.42; *p* = 0.006), and hydrocephalus (OR 1.99, 95% CI 1.22–3.25; *p* = 0.006) remained independently associated with poor neurological outcomes at discharge (Table 4).

After additional adjustment for hospital, the associations of decreased muscle strength, advanced MRC stage, low serum albumin, and hydrocephalus remained significant, whereas the association with elevated white blood cell count was attenuated and no longer reached statistical significance (Supplementary Table S2). These findings indicated that the associations of the major clinical factors were generally robust to adjustment for potential center effects, whereas the association with elevated white blood cell count was less robust and should be interpreted cautiously.

### 3.6. Exploratory Analysis of the Associations of Intensified Treatment and Other Clinical Factors with Neurological Outcomes

Propensity score matching (PSM) was performed on the patient data using intensified treatment exposure as the grouping variable. The propensity score model included hospital, MRC stage, hydrocephalus, muscle strength decline, and albumin level as matching variables. Nearest-neighbor 1:1 matching was performed using a caliper of 0.2 × SD of the logit of the propensity score, without replacement. After matching, 154 patients were included, with 77 patients in each treatment group. The standardized mean difference (SMD) for each covariate was lower than 0.2, indicating a good balance of baseline characteristics between groups (Table 5; Supplementary Figures S1 and S2). After PSM, the matched groups were well balanced with respect to baseline disease severity and treatment center, thereby providing a more balanced comparison for further evaluating the relationship between intensified treatment drugs and neurological outcomes.

In the matched cohort, binary logistic regression analysis was performed to evaluate the association between intensified treatment and neurological outcomes (Table 6 and Table 7). Before matching, intensified treatment was associated with neurological outcomes (OR = 2.04, 95% CI 1.04–4.01, *p* = 0.038), and linezolid and fluoroquinolone use was more frequent among patients with unfavorable neurological outcomes. After treatment-exposure-based PSM, however, intensified treatment was no longer significantly associated with neurological outcomes (OR = 1.11, 95% CI 0.46–2.69, *p* = 0.821). Similarly, no significant associations were observed between individual intensified treatment drugs and neurological outcomes in the matched cohort. Consistent findings were observed in the exploratory outcome-based matched cohort (Supplementary Tables S3 and S4).

In the exploratory outcome-based matched cohort, univariable logistic regression analysis was performed to examine the potential associations between various clinical factors and neurological outcomes (Table 8). The presence of diabetes mellitus showed a potential association with adverse neurological outcomes (OR 2.97, 95% CI 1.11–7.94, *p* = 0.03).

To further explore this association, an additional multivariable logistic regression analysis was performed in the exploratory outcome-based matched cohort, with diabetes mellitus as the variable of interest and age, MRC disease stage, muscle weakness, hydrocephalus, serum albumin, white blood cell count, and intensified treatment as covariates. After adjustment for these potential confounding factors, diabetes mellitus remained potentially associated with adverse neurological outcomes (OR 3.05, 95% CI 1.13–8.21, *p* = 0.027; Supplementary Table S5). However, this finding should be interpreted cautiously as an exploratory association and requires further validation.

### 3.7. Sensitivity Analysis Restricted to Patients with Confirmed or Highly Suspected TBM

After excluding 39 patients with suspected TBM, 479 patients with confirmed or highly suspected TBM were included in the sensitivity analysis. The results were consistent with the primary analysis. Muscle weakness, advanced MRC stage, elevated white blood cell count, serum albumin < 30 g/L, and hydrocephalus remained significantly associated with unfavorable neurological outcomes (Supplementary Table S6). After propensity score matching for intensified treatment exposure (Supplementary Table S7), neither intensified treatment nor individual intensified drugs were significantly associated with neurological outcomes (Supplementary Table S8). These findings further supported the robustness of the primary results.

## 4. Discussion

Tuberculous meningitis (TBM) is associated with high mortality and substantial neurological disability, imposing a considerable socioeconomic burden. Early risk stratification is therefore urgently needed [17,18]. By analyzing the severity of the disease, individual factors, and the impact of drugs on prognosis, we identified factors associated with poor neurological outcomes in TBM, providing a basis for clinical risk assessment and individualized management.

The MRC grading system has served as a cornerstone for TBM stratification for over seven decades. Our findings further support the clinical value of the MRC grading system. However, our analysis suggests that reliance on MRC grading alone may be insufficient for accurately predicting neurological outcomes. Beyond disease severity, hydrocephalus, decreased muscle strength, and serum albumin levels < 30 g/L were also identified as independent risk factors for adverse neurological prognosis.

Hydrocephalus is closely associated with poor prognoses in TBM patients, particularly those with higher MRC grades. Our study reaffirms these findings [19]. The inflammatory response in the subarachnoid space due to Mycobacterium tuberculosis infection, characterized by gelatinous exudate obstructing the cerebrospinal fluid pathway, can initiate a cascade of events leading to increased intracranial pressure and ischemic injury, ultimately resulting in unfavorable outcomes [20]. Head CT/MRI serves as a dependable modality for diagnosing and assessing the severity of hydrocephalus in TBM patients [21]. Medical personnel should vigilantly monitor imaging findings, particularly indications of hydrocephalus, in stage II/III tuberculous meningitis cases. In our investigation, standard treatments for hydrocephalus included anti-tuberculosis drugs, diuretics, and corticosteroids. Pharmacological interventions can effectively alleviate mild-to-moderate hydrocephalus. However, in cases where patients are unresponsive to drug therapy, prompt surgical intervention is necessary to mitigate damage to brain parenchyma [19].

In this study, albumin levels below 30g/L emerge as an independent risk factor for unfavorable neurological outcomes in TBM patients. This finding aligns with prior studies highlighting the link between low albumin levels and adverse tuberculosis prognosis [22,23]. Serum albumin concentration serves as a crucial marker for assessing nutritional status and potentially mirrors patients’ preclinical health and overall nutritional well-being [24]. Moreover, reduced albumin levels (<30 g/L) can modify protein binding rates and the in vivo pharmacokinetics of essential anti-tuberculosis medications like rifampicin and linezolid, impacting their therapeutic efficacy [25]. Hence, it is imperative to regularly monitor albumin levels, conduct comprehensive nutritional assessments, and promptly administer personalized nutritional support to enhance patient outcomes in clinical settings.

The multivariable analysis reveals that reduced muscle strength serves as an independent risk factor for unfavorable neurological outcomes in patients with TBM. Muscle weakness typically signifies evident neurological involvement. The inflammatory response triggered by Mycobacterium tuberculosis infection results in occlusive vasculitis, culminating in cerebral ischemia and infarction. In severe instances, it may directly affect the brain parenchyma [26]. With the escalation of the inflammatory response and the advancement of intracranial lesions, patients become more susceptible to enduring motor impairment, impacting their long-term quality of life. Prior research has demonstrated a close association between neurological impairments, the severity of TBM, and adverse clinical consequences [27]. Hence, healthcare providers must prioritize the ongoing evaluation of changes in muscle strength during clinical care, promptly identify high-risk individuals, and initiate anti-tuberculosis therapy expeditiously to enhance patient prognosis.

Regarding pharmacological intervention, our initial raw data suggested an association between the use of linezolid or fluoroquinolones and poor outcomes. However, after employing PSM to adjust for baseline severity, this association vanished. This suggests a “severity bias,” where clinicians are more likely to prescribe intensified regimens to the most critically ill patients. Our findings align with current evidence indicating that while intensified therapy is recommended for high-risk cases, it does not inherently guarantee improved neurological outcomes compared to standard regimens [28,29]. Consequently, the decision to intensify treatment should be driven by individualized risk assessment rather than a universal application of second-line drugs.

In an exploratory outcome-based matched comparison, diabetes mellitus showed a potential association with unfavorable neurological outcomes. However, this finding is insufficient to establish diabetes as an independent prognostic factor. Further prospective studies are necessary for validation. Previous research consistently shows that diabetes significantly elevates the risks of treatment failure, death, and recurrence in tuberculosis patients. Maintaining good blood glucose control (HbA1c < 7%) correlates with improved clinical outcomes, whereas poor control is linked to a worse prognosis [30,31,32]. Mechanistically, diabetes heightens susceptibility to Mycobacterium tuberculosis, compromises protective immunity via hyperglycemia-induced oxidative stress, disrupts the blood–brain barrier, and impairs macrophage function, thereby impeding tuberculosis management and treatment responses [33,34]. Therefore, diabetes mellitus may represent an important comorbidity requiring clinical attention in patients with tuberculous meningitis.

Although a center-level association was observed, the underlying mechanisms remain unclear. Differences between centers in patient characteristics, referral patterns, clinical management, treatment strategies, and supportive care may have contributed to this finding. In addition, given the 11-year study period, temporal changes in case mix, diagnostic techniques, and clinical documentation practices may also have influenced the observed association. To assess the potential impact of these temporal changes, we performed a sensitivity analysis adjusting for admission period. Adjustment for admission period did not materially alter the associations of the major clinical factors (Supplementary Tables S9 and S10). Therefore, the observed hospital-related association should be interpreted as a potential center effect rather than a patient-level biological predictor and should not be interpreted as a comparison of healthcare quality between institutions.

Several limitations warrant acknowledgment: (1) Lack of long-term outcomes: Neurological outcomes were assessed only at discharge, and longer-term functional recovery was not evaluated. Therefore, further prospective studies with long-term follow-up are needed to better evaluate the long-term neurological outcomes of TBM patients. (2) Retrospective bias: The reliance on historical medical records introduces potential record bias and limits the standardization of certain variables. Therefore, the potential impact of intensified treatment timing and duration on neurological outcomes could not be fully evaluated. This limitation restricts causal inference from our findings. (3) Universality: Despite adopting a two-center approach, variations in clinical pathways may lead to center bias. (4) Residual confounding factors: Although we adjust for known confounding factors as thoroughly as possible through multivariate analysis, unmeasured or unquantifiable clinical variables may still influence the results. (5) Impact of missing data: As this was a retrospective study, missing data were present for some clinical variables. Although appropriate strategies were implemented to reduce the potential impact of missing data, residual selection bias or information bias related to missing data cannot be completely ruled out.

## 5. Conclusions

MRC stage II/III, decreased muscle strength, hydrocephalus, and serum albumin levels below 30 g/L were independently associated with poor neurological outcomes in tuberculous meningitis. After adjusting for disease severity and patients’ underlying conditions, intensified therapy was not significantly associated with neurological outcome at discharge. An exploratory association between diabetes mellitus and poor neurological outcomes was also observed, necessitating further verification. It is essential to enhance the precise stratified assessment of disease severity and prioritize nutrition and individualized comprehensive management to improve neurological function outcomes.

## Figures and Tables

**Table 1:** Comparison of ts for Variable Selection in LASSO RegresDisease Severity Between TBM Patients with Different Prognoses.

Variables	Total (*n* = 518)	Favorable Prognosis (*n* = 395)	Unfavorable Prognosis (*n* = 123)	|SMD|/*p*
TBM diagnostic certainty, *n* (%)				0.210
Confirmed	311 (60.04%)	237 (60.00%)	74 (60.16%)	
Highly suspected	168 (32.43%)	124 (31.39%)	44 (35.77%)	
Suspected	39 (7.53%)	34 (8.61%)	5 (4.07%)	
MRC Disease Stage, *n* (%)				<0.001
Stage I	172 (33.20%)	160 (40.51%)	12 (9.76%)	
Stage II	276 (53.28%)	202 (51.14%)	74 (60.16%)	
Stage III	70(13.51%)	33(8.35%)	37 (30.08%)	
GCS score, M (Q1, Q3)	15.00 (13.00–15.00)	15.00 (14.00–15.00)	14.00 (9.00–15.00)	0.499 *
Concurrent Pulmonary Tuberculosis, *n* (%)	253 (48.84%)	196 (49.62%)	57 (46.34%)	0.066
Drug Resistance Testing Performed, *n* (%)	81 (15.64)	55 (13.92)	26 (21.14)	0.199
Drug-resistant TB, *n* (%)	16 (3.09%)	10 (2.53%)	6 (4.88%)	0.109
Length of Hospital Stay (Days), M (Q_1_,Q_3_)	30.00 (18.00–46.00)	30.00 (18.00–47.00)	30.00 (17.00–43.00)	0.157
Treatment duration, median (Q1, Q3), days	44.00 (28.00, 74.75)	44.00 (29.00, 77.00)	44.00 (22.00, 69.00)	0.074
Treatment delay, median (Q1, Q3), days	18.00 (11.00, 36.00)	18.00 (11.00, 36.50)	17.00 (9.50, 36.00)	0.401
Clinical manifestations, *n* (%)				
Fever > 38.5 °C	416 (80.31%)	322 (81.52%)	94 (76.42%)	0.120
Cough > 2 weeks	114 (22.01%)	93 (23.54%)	21 (17.07%)	0.172
Weight Loss > 5%	108 (20.85%)	84 (21.27%)	24 (19.51%)	0.044
Vomiting	161 (31.08%)	130 (32.91%)	31 (25.20%)	0.178
Seizures	51 (9.85%)	33 (8.35%)	18 (14.63%)	0.178
Disturbance of Consciousness	234 (45.17%)	155 (39.24%)	79 (64.23%)	0.521 *
Meningeal Irritation signs	238 (45.95%)	179 (45.32%)	59 (47.97%)	0.053
Pathological Reflexes	98 (18.92%)	73 (18.48%)	25 (20.33%)	0.096
Cranial Nerve Palsy	49 (9.46%)	30 (7.59%)	19 (15.45%)	0.217 *
Muscle Weakness	104 (20.08%)	56 (14.18%)	48 (39.02%)	0.509 *
Abnormal Muscle Tone	43 (8.30%)	24 (6.08%)	19 (15.45%)	0.259 *
Imaging findings, *n* (%)				
Cerebral Infarction	213 (41.12%)	158 (40.00%)	55 (44.72%)	0.095
Tuberculoma	44 (8.49%)	37 (9.37%)	7 (5.69%)	0.159
Hydrocephalus	136 (26.25%)	88 (22.28%)	48 (39.02%)	0.343 *
Intracranial Hemorrhage	8 (1.54%)	4 (1.01%)	4 (3.25%)	0.126
Meningeal Enhancement	141 (27.22%)	106 (26.84%)	35 (28.46%)	0.036
External Ventricular Drainage	14 (2.70)	8 (2.03)	6 (4.88)	0.176

Note: TBM, tuberculous meningitis; MRC, Medical Research Council staging criteria for tuberculous meningitis; GCS, Glasgow Coma Scale; M, median; Q_1_, first quartile; Q_3_, third quartile. Consciousness disturbance refers to impairment of alertness or awareness, ranging from drowsiness to coma. Cranial nerve palsy refers to dysfunction of one or more cranial nerves, presenting as diplopia, facial asymmetry, dysphagia, or impaired ocular movement. Muscle weakness refers to decreased voluntary muscle strength detected on neurological examination. Abnormal muscle tone refers to abnormal resistance to passive movement, including increased (spasticity or rigidity) or decreased muscle tone. Standardized mean differences (SMDs) were used for continuous variables and categorical variables with two categories, whereas *p* values were used for categorical variables with three or more categories. * SMD > 0.2 was considered to indicate meaningful imbalance between groups.

**Table 2:** Comparison of Individual Factors and Laboratory Tests Between TBM Patients with Different Prognoses.

Variables	Total (*n* = 518)	Favorable Prognosis (*n* = 395)	Unfavorable Prognosis (*n* = 123)	|SMD|/*p*
Hospital, *n* (%)				
Public Health Hospital	334 (64.48%)	239 (60.51%)	95 (77.24%)	0.399 *
Red Cross Hospital	184 (35.52%)	156 (39.49%)	28 (22.76%)	0.399 *
Male, *n* (%)	325 (62.74%)	243 (61.52%)	82 (66.67%)	0.109
Age (Years), M (Q_1_,Q_3_)	48.00 (30.00–61.00)	46.00 (29.00–60.00)	53.00 (34.00–63.00)	0.204 *
BMI (kg/m^2^), M (Q_1_,Q_3_)	20.76 (18.15–23.83)	20.76 (18.28–23.66)	20.76 (18.01–24.16)	0.033
Smoking History, *n* (%)	95 (18.34%)	77 (19.49%)	18 (14.63%)	0.137
Drinking History, *n* (%)	67 (12.93%)	49 (12.41%)	18 (14.63%)	0.063
Hypertension, *n* (%)	79 (15.25%)	54 (13.67%)	25 (20.33%)	0.017
Diabetes Mellitus, *n* (%)	56 (10.81%)	39 (9.87%)	17 (13.82%)	0.114
HIV, *n* (%)	8 (1.54%)	5 (1.27%)	3 (2.44%)	0.076
Hematological Examinations				
Leukocyte Count (×10^9^/L), M (Q_1_,Q_3_)	6.70 (4.89–8.84)	6.56 (4.88–8.50)	7.59 (5.12–10.20)	0.314 *
Red Blood Cell Count (10^12^/L), mean ± SD	4.11 ± 0.68	4.14 ± 0.67	4.02 ± 0.70	0.169
Hemoglobin (g/L), mean ± SD	122.32 ± 20.60	122.85 ± 20.03	120.60 ± 22.32	0.101
Platelet Count (10^9^/L), M (Q_1_,Q_3_)	213.00 (162.25–264.00)	213.00 (165.00–263.00)	213.00 (150.00–267.00)	0.002
Neutrophil-to-Lymphocyte Ratio (NLR), M (Q_1_,Q_3_)	6.83 (3.58, 12.01)	6.50 (3.58, 11.29)	8.94 (3.59, 18.26)	0.261 *
Alanine Aminotransferase (U/L), Median (Q_1_, Q_3_)	19.50 (12.00–36.00)	19.00 (12.00–35.00)	22.00 (13.00–41.50)	0.100
Aspartate Aminotransferase (U/L), Median (Q_1_, Q_3_)	20.00 (15.00–31.00)	19.00 (15.00–30.00)	24.00 (16.00–34.50)	0.139
Total Bilirubin (μmol/L), Median (Q_1_, Q_3_)	12.30 (7.40–18.50)	11.60 (7.35–18.60)	13.00 (8.00–18.10)	0.038
Direct Bilirubin (μmol/L), Median (Q_1_, Q_3_)	5.80 (3.40–9.55)	5.60 (3.30–9.30)	6.40 (4.00–9.85)	0.174
Indirect Bilirubin (μmol/L), Median (Q_1_, Q_3_)	6.00 (3.70–9.40)	6.00 (3.70–9.84)	5.90 (3.70–8.50)	0.214 *
Total Protein (g/L), Median (Q_1_, Q_3_)	64.90 (59.35–70.30)	65.20 (59.90–70.40)	63.50 (58.05–68.65)	0.146
Albumin (g/L), Median (Q_1_, Q_3_)	37.16 (32.70–40.73)	37.60 (33.33–40.95)	35.70 (30.95–38.92)	0.349 *
Serum Potassium (mmol/L), Median (Q_1_, Q_3_)	3.90 (3.51–4.20)	3.86 (3.50–4.19)	4.00 (3.65–4.28)	0.287 *
Serum Sodium (mmol/L), Median (Q_1_, Q_3_)	134.55 (130.00–139.00)	135.00 (130.35–138.65)	133.90 (129.05–140.00)	0.046
Serum Creatinine (μmol/L), Median (Q_1_, Q_3_)	56.00 (44.40–70.38)	56.90 (45.30–70.34)	53.60 (40.75–70.56)	0.073
Cerebrospinal Fluid (CSF) Analysis				
Intracranial Pressure (mmH_2_O), Median (Q_1_, Q_3_)	180.00 (130.00–260.00)	180.00 (120.00–260.00)	180.00 (130.00–252.50)	0.025
White Blood Cell Count (×10^6^/L), Median (Q_1_, Q_3_)	80.00 (20.00–191.50)	83.00 (20.00–191.00)	70.00 (15.00–197.00)	0.064
Lactate Dehydrogenase (U/L), Median (Q_1_, Q_3_)	49.00 (31.00–80.75)	46.00 (30.00–70.00)	64.00 (39.00–113.50)	0.285 *
Adenosine Deaminase (U/L), Median (Q_1_, Q_3_)	5.00 (2.00–9.44)	5.00 (2.00–9.00)	5.30 (2.00–11.92)	0.158
Chloride (mmol/L), Median (Q_1_, Q_3_)	115.25 (109.00–120.40)	116.00 (109.10–120.60)	114.60 (108.07–120.15)	0.190
Glucose Ratio, Median (Q_1_, Q_3_)	0.39 (0.27–0.54)	0.40 (0.28–0.53)	0.36 (0.25–0.55)	0.001
CSF Protein (g/L), Median (Q_1_, Q_3_)	1.19 (0.69–2.11)	1.14 (0.66–1.98)	1.34 (0.72–2.83)	0.120

Note: TBM, Tuberculous Meningitis; BMI, Body Mass Index; HIV, Human Immunodeficiency Virus; M, Median; Q_1_, First Quartile; Q_3_, Third Quartile; mean ± SD, Mean ± Standard Deviation. All laboratory indicators were measured at admission; CSF examination indicators were derived from the first lumbar puncture results. Standardized mean differences (SMDs) were used for continuous variables and categorical variables with two categories, whereas *p* values were used for categorical variables with three or more categories. * SMD > 0.2 was considered to indicate meaningful imbalance between groups.

**Table 3:** Comparison of Medication Use Between TBM Patients with Different Prognoses.

Variables	Total (*n* = 518)	Favorable Prognosis (*n* = 395)	Unfavorable Prognosis (*n* = 123)	|SMD|/*p*
INH, *n* (%)	515 (99.42)	393 (99.49)	122 (99.19)	0.034
Rifamycins, *n* (%)	496 (95.75)	378 (95.70)	118 (95.93)	0.074
PZA, *n* (%)	475 (91.7)	361 (91.39)	114 (92.68)	0.087
EMB, *n* (%)	487 (94.02)	371 (93.92)	116 (94.31)	0.017
LZD, *n* (%)	233 (44.98)	161 (40.76)	72 (58.54)	0.361 *
FQs, *n* (%)	405 (78.19)	297 (75.19)	108 (87.80)	0.386 *
Amk, *n* (%)	111 (21.43)	81 (20.51)	30 (24.39)	0.090
Enhance drug quantity				0.317
1	37 (7.14)	30 (7.59)	7 (5.69)	
2	352 (67.95)	264 (66.84)	88 (71.54)	
3	52 (10.04)	37 (9.37)	15 (12.20)	
Mannitol, *n* (%)	424 (81.85)	320 (81.01)	104 (84.55)	0.098
Corticosteroids, *n* (%)	476 (91.89)	363 (91.90)	113 (91.87)	0.001

Note: TBM, Tuberculous Meningitis; INH, Isoniazid; PZA, Pyrazinamide; EMB, Ethambutol; LZD, Linezolid; FQs, Fluoroquinolones; Amk, Amikacin. Standardized mean differences (SMDs) were used for continuous variables and categorical variables with two categories, whereas *p* values were used for categorical variables with three or more categories. * SMD > 0.2 was considered to indicate meaningful imbalance between groups.

**Table 4:** Independent Factors Associated With Poor Neurological Outcomes in TBM.

Variables	β	S.E	Z	*p*	OR (95%CI)
Age > 60	0.15	0.26	0.59	0.557	1.16 (0.70~1.93)
Muscle Weakness	1.29	0.27	4.69	<0.001 *	3.63 (2.12~6.21)
MRC Disease Stage II	0.94	0.36	2.60	0.009 *	2.56 (1.26~5.21)
MRC Disease Stage III	2.32	0.40	5.76	<0.001 *	10.18 (4.62~22.42)
White Blood Cell Count > 11 × 10^9^/L	0.75	0.32	2.34	0.019 *	2.13 (1.13~4.00)
Serum Albumin < 30 g/L	0.87	0.31	2.77	0.006 *	2.39 (1.29~4.42)
Hydrocephalus	0.69	0.25	2.77	0.006 *	1.99 (1.22~3.25)

Note: OR, Odds Ratio; CI, Confidence Interval; S.E, Standard Error; β, Regression Coefficient. MRC, Medical Research Council; * indicates statistical significance (*p* < 0.05).

**Table 5:** Baseline Characteristics Before and After Propensity Score Matching According to Intensified Treatment Exposure.

Variable	Before PSM						After PSM					
	Total (*n* = 518)	Control Group (*n* = 77)	Treatment Group (*n* = 441)	Statistic	*p*	SMD	Total (*n* = 154)	Control Group (*n* = 77)	Treatment Group (*n* = 77)	Statistic	*p*	SMD
Hospital, *n* (%)				χ^2^ = 54.664	<0.001					χ^2^ = 0.000	1.000	
Public Health Hospital	334 (64.48)	21 (27.27)	313 (70.98)			0.963	42 (27.27)	21 (27.27)	21 (27.27)			0.000
Red Cross Hospital	184 (35.52)	56 (72.73)	128 (29.02)			−0.963	112 (72.73)	56 (72.73)	56 (72.73)			0.000
Muscle Strength, *n* (%)				χ^2^ = 0.202	0.653					χ^2^ = 0.166	0.684	
Impaired	104 (20.08)	14 (18.18)	90 (20.41)			0.055	30 (19.48)	14 (18.18)	16 (20.78)			0.064
Normal	414 (79.92)	63 (81.82)	351 (79.59)			−0.055	124 (80.52)	63 (81.82)	61 (79.22)			−0.064
MRC Disease Stage, *n* (%)				χ^2^ = 11.110	0.004					-	0.902	
Stage I	172 (33.2)	34 (44.16)	138 (31.29)			−0.272	70 (45.45)	34 (44.16)	36 (46.75)			0.052
Stage II	276 (53.28)	41 (53.25)	235 (53.29)			−0.004	81 (52.6)	41 (53.25)	40 (51.95)			−0.026
Stage III	70 (13.51)	2 (2.6)	68 (15.42)			0.355	3 (1.95)	2 (2.60)	1 (1.30)			−0.115
Serum Albumin, *n* (%)				χ^2^ = 1.401	0.237					χ^2^ = 1.501	0.221	
<30 g/L	69 (13.32)	7 (9.09)	62 (14.06)			0.143	19 (12.34)	7 (9.09)	12 (15.58)			0.179
≥30 g/L	449 (86.68)	70 (90.91)	379 (85.94)			−0.143	135 (87.66)	70 (90.91)	65 (84.42)			−0.179
Hydrocephalus, *n* (%)				χ^2^ = 5.319	0.021					χ^2^ = 0.496	0.481	
Absent	382 (73.75)	65 (84.42)	317 (71.88)			−0.279	133 (86.36)	65 (84.42)	68 (88.31)			0.121
Present	136 (26.25)	12 (15.58)	124 (28.12)			0.279	21 (13.64)	12 (15.58)	9 (11.69)			−0.121

Note: PSM, Propensity Score Matching; SMD, Standardized Mean Difference; MRC, Medical Research Council Staging Criteria for Tuberculous Meningitis. SMD is used to assess the balance of covariates before and after matching; SMD < 0.2 indicates that the baseline characteristics between the groups are well balanced.

**Table 6:** Univariable Logistic Regression Analysis of Intensified Treatment Drugs and Neurological Functional Outcomes in the Matched Cohort Based on Intensified Treatment Exposure.

Variables	β	S.E	Z	*p*	OR (95% CI)
Intensified Treatment	0.10	0.45	0.23	0.821	1.11 (0.46~2.69)
Linezolid (Lzd)	0.02	0.55	0.04	0.969	1.02 (0.35~2.99)
Amikacin (Amk)	−1.19	1.05	−1.13	0.260	0.30 (0.04~2.41)
Fluoroquinolone (FQs)	0.14	0.45	0.32	0.752	1.15 (0.47~2.80)
Number of intensified drugs = 1	0.98	0.73	1.34	0.181	2.66 (0.63~11.13)
Number of intensified drugs = 2	−0.49	0.46	−1.08	0.278	0.61 (0.25~1.49)
Number of intensified drugs = 3	0.41	0.61	0.68	0.498	1.51 (0.46~5.02)
Corticosteroids	−0.49	0.69	−0.71	0.478	0.61 (0.16~2.38)
Mannitol	−0.26	0.52	−0.50	0.616	0.77 (0.28~2.14)

Note: OR, Odds Ratio; CI, Confidence Interval; S.E, Standard Error; β, Regression Coefficient.

**Table 7:** Association Between Intensified Treatment and Clinical Outcomes in Patients With Tuberculous Meningitis Before and After Propensity Score Matching.

Clinical Outcome Measure	Overall	Control Group	Intensified Treatment Group	*p* Value	OR (95% CI)
Before matching	*n* = 518	*n* = 77	*n* = 441	—	—
Modified Rankin Scale (mRS)	1.00 (0.00, 2.00)	0.00 (0.00, 1.00)	1.00 (0.00, 3.00)	0.038 *	2.04 (1.04–4.01)
After matching	*n* = 154	*n* = 77	*n* = 77	—	—
Modified Rankin Scale (mRS)	0.00 (0.00, 1.00)	0.00 (0.00, 1.00)	0.00 (0.00, 1.00)	0.821	1.11 (0.46–2.69)

* indicates statistical significance (*p* < 0.05).

**Table 8:** Univariable Logistic Regression Analysis of Other Clinical Factors and Neurological Functional Outcomes in the Exploratory Outcome-based Matched Cohort.

Variables	β	S.E	Z	*p*	OR (95%CI)
Diabetes	1.09	0.50	2.18	0.030 *	2.97 (1.11~7.94)

Note: OR, Odds Ratio; CI, Confidence Interval; S.E, Standard Error; β, Regression Coefficient. * indicates statistical significance (*p* < 0.05).

## Data Availability

The datasets generated and/or analyzed during the current study are available from the corresponding author on reasonable request.

## Supplementary Materials

The following supporting information can be downloaded at: https://www.mdpi.com/article/10.3390/tropicalmed11090259/s1. Table S1: Characteristics of Treatment-Related Adverse Events. Table S2: Independent Factors Associated With Poor Neurological Outcomes in Tuberculous Meningitis. Table S3: Comparison of SMD of Patient Characteristics Before and After PSM in the Exploratory Outcome-Based Matched Cohort. Table S4: Univariable Logistic Regression Analysis of Intensified Treatment Drugs and Neurological Functional Outcomes in the Exploratory Outcome-Based Matched Cohort. Table S5: Multivariable Logistic Regression Analysis of Factors Associated With Poor Neurological Outcomes in the Exploratory Outcome-Based Matched Cohort. Table S6: Sensitivity Analysis of Factors Associated With Poor Neurological Outcomes at Discharge Among Patients With Confirmed or Highly Suspected TBM. Table S7: Baseline Characteristics Before and After Propensity Score Matching According to Intensified Treatment Exposure Among Patients With Confirmed or Highly Suspected TBM. Table S8: Univariable Logistic Regression Analysis of the Associations Between Intensified Treatment Drugs and Neurological Functional Outcomes in the Matched Cohort of Patients With Confirmed or Highly Suspected TBM. Table S9: Temporal trends in diagnostic characteristics, treatment patterns, and clinical features of tuberculous meningitis patients from 2012 to 2023. Table S10: Sensitivity Analysis of Factors Associated With Unfavorable Neurological Outcomes After Adjustment for Admission Period. Figure S1: Covariate Balance Before and After 1:1 Propensity Score Matching According to Intensified Treatment Exposure. Figure S2: Propensity score distribution before and after 1:1 nearest-neighbor matching to intensified treatment exposure.
