# Supplementary material for: Risk Factors for Poor Neurological Outcomes at Discharge in Tuberculous Meningitis: Disease Severity, Host Factors, and Drug Therapy
Source: Trop Med Infect Dis. 2026 Sep 14;11(9):259. doi: 10.3390/tropicalmed11090259 (PMC13611471; doi:10.3390/tropicalmed11090259)
Supplement: Supplementary file 1 [file tropicalmed-11-00259-s001.zip › tropicalmed-4360981-supplementary.pdf]

Supplementary Table S1. Characteristics of Treatment-related Adverse Events.

**Variables**	**Total (*n* = 518)**	**Intensified Treatment (*n*=441)**	**Standard treatment(*n*=77)**	**P value**
Liver injury, *n* (%)	85 (16.41)	77 (17.46)	8 (10.39)	0.122
Renal injury, *n* (%)	5 (0.97)	5 (1.13)	0 (0.00)	1.000
Rash, *n* (%)	33 (6.37)	33 (7.48)	0 (0.00)	0.026*
Leukopenia, *n* (%)	13 (2.51)	13 (2.95)	0 (0.00)	0.258
Visual impairment, *n* (%)	19 (3.67)	17 (3.85)	2 (2.60)	0.831
Anemia, *n* (%)	8 (1.54)	8 (1.81)	0 (0.00)	0.490
Thrombocytopenia, *n* (%)	13 (2.51)	12 (2.72)	1 (1.30)	0.733
Peripheral numbness, *n* (%)	5 (0.97)	4 (0.91)	1 (1.30)	0.554

* indicates statistical significance (P<0.05)

Supplementary Table S2. Independent Factors Associated With Poor Neurological Outcomes in Tuberculous Meningitis.

**Variables**	**β**	**S.E**	**Z**	**P**	**OR (95%CI)**
Age >60	0.2509	0.2589	0.9693	0.3324	1.29 (0.77-2.14)
Hospital (Red Cross Hospital)	-0.9181	0.275	-3.3378	<0.001*	0.40 (0.23-0.69)
Muscle Weakness	1.3594	0.2849	4.7713	<0.001*	3.89 (2.22-6.82)
MRC Disease Stage II	0.9051	0.3656	2.4754	0.0133*	2.47 (1.21-5.07)
MRC Disease Stage III	2.2382	0.4078	5.4886	<0.001*	9.38 (4.21-20.89)
White Blood Cell Count $>11\times10^9$/L	0.6052	0.3328	1.8189	0.0689	1.83 (0.95-3.52)
Serum Albumin <30 g/L	1.0094	0.3267	3.0894	0.0020*	2.74 (1.44–5.21)
Hydrocephalus	0.7049	0.2543	2.7722	0.0056*	2.02 (1.23-3.33)

* indicates statistical significance (P<0.05)

Supplementary Table S3 Comparison of SMD of Patient Characteristics Before and After PSM in the Exploratory Outcome-Based Matched Cohort

**Variable**	**Before PSM**						**After PSM**					
	**Total (*n* = 518)**	**Favorable Prognosis (*n*=395)**	**Unfavorable Prognosis (*n*=123)**	**Statistic**	**P**	**SMD**	**Total (*n* = 206)**	**Favorable Prognosis (*n*=103)**	**Unfavorable Prognosis (*n*=103)**	**Statistic**	**P**	**SMD**
Hospital, *n* (%)				χ^2=11.461	<.001					χ^2=0.000	1.000	
Public Health Hospital	334 (64.48)	239 (60.51)	95 (77.24)			0.399	152 (73.79)	76 (73.79)	76 (73.79)			0.000
Red Cross Hospital	184 (35.52)	156 (39.49)	28 (22.76)			-0.399	54 (26.21)	27 (26.21)	27 (26.21)			0.000
Sex, *n* (%)				χ^2=1.063	0.302					χ^2=0.553	0.457	
Female	193 (37.26)	152 (38.48)	41 (33.33)			-0.109	67 (32.52)	36 (34.95)	31 (30.10)			-0.09
Male	325 (62.74)	243 (61.52)	82 (66.67)			0.109	139 (67.48)	67 (65.05)	72 (69.90)			0.09
Age, *n* (%)				χ^2=1.352	0.245					χ^2=0.023	0.879	
>60	135 (26.06)	98 (24.81)	37 (30.08)			0.115	61 (29.61)	30 (29.13)	31 (30.10)			0.021
≤60	383 (73.94)	297 (75.19)	86 (69.92)			-0.115	145 (70.39)	73 (70.87)	72 (69.90)			-0.021
Muscle Strength, *n* (%)				χ^2=36.087	<.001					χ^2=0.085	0.770	
Impaired	104 (20.08)	56 (14.18)	48 (39.02)			0.509	72 (34.95)	37 (35.92)	35 (33.98)			-0.041
Normal	414 (79.92)	339 (85.82)	75 (60.98)			-0.509	134 (65.05)	66 (64.08)	68 (66.02)			0.041
MRC Disease Stage, *n* (%)				χ^2=61.279	<.001					χ^2=0.131	0.937	
Stage I	172 (33.2)	160 (40.51)	12 (9.76)			-1.045	23 (11.17)	12 (11.65)	11 (10.68)			-0.031

Stage II	276 (53.28)	202 (51.14)	74 (60.16)			0.189	133 (64.56)	67 (65.05)	66 (64.08)			-0.020
Stage III	70 (13.51)	33 (8.35)	37 (30.08)			0.474	50 (24.27)	24 (23.30)	26 (25.24)			0.045
Serum Albumin, *n* (%)				χ^2=5.356	0.021					χ^2=0.000	1.000	
<30 g/L	69 (13.32)	45 (11.39)	24 (19.51)			0.205	30 (14.56)	15 (14.56)	15 (14.56)			0.000
≥30 g/L	449 (86.68)	350 (88.61)	99 (80.49)			-0.205	176 (85.44)	88 (85.44)	88 (85.44)			0.000
Hydrocephalus, *n* (%)				χ^2=13.585	<.001					χ^2=0.084	0.771	
Absent	382 (73.75)	307 (77.72)	75 (60.98)			-0.343	132 (64.08)	65 (63.11)	67 (65.05)			0.041
Present	136 (26.25)	88 (22.28)	48 (39.02)			0.343	74 (35.92)	38 (36.89)	36 (34.95)			-0.041

Note: PSM, Propensity Score Matching; SMD, Standardized Mean Difference; TBM, Tuberculous Meningitis; MRC, Medical Research Council Staging Criteria for Tuberculous Meningitis. SMD is used to assess the balance of covariates before and after matching; SMD<0.2 indicates that the baseline characteristics between the groups are well balanced.

Supplementary Table S4 Univariable Logistic Regression Analysis of Intensified Treatment Drugs and Neurological Functional Outcomes in the Exploratory Outcome-Based Matched Cohort

**Variables**	**β**	**S.E**	**Z**	**P**	**OR (95% CI)**
Linezolid (LZD)	0.08	0.28	0.28	0.779	1.08 (0.62 ~ 1.88)
Amikacin (Amk)	-0.15	0.32	-0.48	0.633	0.86 (0.46 ~ 1.60)
Fluoroquinolone(FQs)	0.15	0.39	0.38	0.701	1.16 (0.54 ~ 2.47)
Number of intensified drugs = 1	-0.36	0.60	-0.59	0.554	0.70 (0.21 ~ 2.28)
Number of intensified drugs = 2	0.40	0.30	1.34	0.182	1.49 (0.83 ~ 2.68)
Number of intensified drugs = 3	-0.38	0.39	-0.97	0.332	0.68 (0.32 ~ 1.48)
Corticosteroids	-0.13	0.51	-0.25	0.800	0.88 (0.33 ~ 2.38)
Mannitol	-0.40	0.41	-1.00	0.319	0.67 (0.30 ~ 1.48)

Note: OR, Odds Ratio; CI, Confidence Interval; S.E, Standard Error; β, Regression Coefficient.

Supplementary Table S5. Multivariable Logistic Regression Analysis of Factors Associated With Poor Neurological Outcomes in the Exploratory Outcome-Based Matched Cohort

**Variables**	**β**	**S.E**	**Z**	**P**	**OR (95%CI)**
Muscle Strength	0.08	0.35	0.24	0.811	1.09 (0.55 ~ 2.14)
MRC Disease Stage					
Stage II	0.07	0.50	0.15	0.883	1.08 (0.41 ~ 2.85)
Stage III	0.14	0.53	0.28	0.783	1.16 (0.41 ~ 3.24)
Hydrocephalus	-0.14	0.31	-0.47	0.641	0.87 (0.48 ~ 1.58)
Intensified Treatment	0.07	0.46	0.15	0.878	1.07 (0.43 ~ 2.67)
Serum Albumin<30 g/L	-0.09	0.41	-0.22	0.827	0.91 (0.41 ~ 2.04)
Diabetes	1.11	0.51	2.20	0.027*	3.05 (1.13 ~ 8.21)

Note: OR, Odds Ratio; CI, Confidence Interval; S.E, Standard Error; β, Regression Coefficient.

Supplementary Table S6. Sensitivity Analysis of Factors Associated With Poor Neurological Outcomes at Discharge Among Patients With Confirmed or Highly Suspected TBM

**Variables**	**β**	**S.E**	**Z**	**P**	**OR (95%CI)**
Age >60	0.23	0.27	0.85	0.395	1.25 (0.75 ~ 2.11)
Muscle Weakness	1.37	0.28	4.87	<.001*	3.93 (2.27 ~ 6.82)
MRC Disease Stage II	0.81	0.37	2.22	0.026*	2.26 (1.10 ~ 4.63)
MRC Disease Stage III	2.16	0.41	5.26	<.001*	8.64 (3.87 ~ 19.29)
White Blood Cell Count $>11\times10^9$/L	0.77	0.34	2.27	0.023*	2.15 (1.11 ~ 4.16)
Serum Albumin <30 g/L	0.85	0.32	2.65	0.008*	2.33 (1.25 ~ 4.35)
Hydrocephalus	0.63	0.25	2.47	0.013*	1.87 (1.14 ~ 3.08)

Note: OR, Odds Ratio; CI, Confidence Interval; S.E, Standard Error; β, Regression Coefficient. MRC, Medical Research Council;* indicates statistical significance (P<0.05).

Supplementary Table S7. Baseline Characteristics Before and After Propensity Score Matching According to Intensified Treatment Exposure Among Patients With Confirmed or Highly Suspected TBM

**Variable**	**Before PSM**						**After PSM**					
	**Total (*n* = 479)**	**Control group (*n* = 77)**	**Treatment group (*n* = 402)**	**Statistic**	**P**	**SMD**	**Total (*n* = 154)**	**Control group (*n* = 77)**	**Treatment group (*n* = 77)**	**Statistic**	**P**	**SMD**
Hospital, *n* (%)				χ^2=45.664	<.001					χ^2=0.032	0.857	
Public Health Hospital	295 (61.59)	21 (27.27)	274 (68.16)			0.878	43 (27.92)	21 (27.27)	22 (28.57)			0.029
Red Cross Hospital	184 (38.41)	56 (72.73)	128 (31.84)			-0.878	111 (72.08)	56 (72.73)	55 (71.43)			-0.029
Muscle Strength, *n* (%)				χ^2=0.243	0.622					χ^2=0.631	0.427	
Normal	382 (79.75)	63 (81.82)	319 (79.35)			-0.061	122 (79.22)	63 (81.82)	59 (76.62)			-0.123
Impaired	97 (20.25)	14 (18.18)	83 (20.65)			0.061	32 (20.78)	14 (18.18)	18 (23.38)			0.123
MRC Disease Stage, *n* (%)				χ^2=13.180	0.001					-	1.000	
Stage I	151 (31.52)	34 (44.16)	117 (29.10)			-0.331	68 (44.16)	34 (44.16)	34 (44.16)			0.000
Stage II	261 (54.49)	41 (53.25)	220 (54.73)			0.030	82 (53.25)	41 (53.25)	41 (53.25)			0.000
Stage III	67 (13.99)	2 (2.60)	65 (16.17)			0.369	4 (2.6)	2 (2.60)	2 (2.60)			0.000
Serum Albumin, *n* (%)				χ^2=1.828	0.176					χ^2=0.084	0.772	
≥30 g/L	412 (86.01)	70 (90.91)	342 (85.07)			-0.164	141 (91.56)	70 (90.91)	71 (92.21)			0.048
<30 g/L	67 (13.99)	7 (9.09)	60 (14.93)			0.164	13 (8.44)	7 (9.09)	6 (7.79)			-0.048
Hydrocephalus, *n* (%)				χ^2=6.788	0.009					χ^2=0.051	0.821	
Absent	346 (72.23)	65 (84.42)	281 (69.90)			-0.316	131 (85.06)	65 (84.42)	66 (85.71)			0.037
Present	133 (27.77)	12 (15.58)	121 (30.10)			0.316	23 (14.94)	12 (15.58)	11 (14.29)			-0.037

Note: PSM, Propensity Score Matching; SMD, Standardized Mean Difference; MRC, Medical Research Council Staging Criteria for Tuberculous Meningitis. SMD is used to assess the balance of covariates before and after matching; SMD<0.2 indicates that the baseline characteristics between the groups are well balance.

Supplementary Table S8. Univariable Logistic Regression Analysis of the Associations Between Intensified Treatment Drugs and Neurological Functional Outcomes in the Matched Cohort of Patients With Confirmed or Highly Suspected TBM

**Variables**	**β**	**S.E**	**Z**	**P**	**OR (95% CI)**
Intensified Treatment	0.29	0.44	0.65	0.513	1.33 (0.56 ~ 3.16)
Linezolid (Lzd)	0.48	0.48	1.01	0.311	1.62 (0.64 ~ 4.14)
Amikacin (Amk)	-0.11	0.67	-0.16	0.873	0.90 (0.24 ~ 3.33)
Fluoroquinolone(FQs)	0.19	0.44	0.42	0.673	1.20 (0.51 ~ 2.84)
Number of intensified drugs = 1	0.15	0.81	0.18	0.856	1.16 (0.24 ~ 5.72)
Number of intensified drugs = 2	-0.12	0.45	-0.26	0.792	0.89 (0.37 ~ 2.13)
Number of intensified drugs = 3	0.30	0.61	0.49	0.625	1.35 (0.41 ~ 4.42)
Corticosteroids	0.07	0.80	0.09	0.931	1.07 (0.22 ~ 5.16)
Mannitol	-0.09	0.55	-0.16	0.870	0.91 (0.31 ~ 2.68)

Note: OR, Odds Ratio; CI, Confidence Interval; S.E, Standard Error; β, Regression Coefficient.

Supplementary Table S9 Temporal trends in diagnostic characteristics, treatment patterns, and clinical features of tuberculous meningitis patients from 2012 to 2023

**Variables**	**Total (*n* = 518)**	**2012–2015 (*n* = 150)**	**2016-2019 (*n* = 193)**	**2020-2023 (*n* = 175)**	**P**
MRC Disease Stage, *n* (%)					0.234
Stage I	172 (33.20)	53 (35.33)	60 (31.09)	59 (33.71)	
Stage II	276 (53.28)	70 (46.67)	111 (57.51)	95 (54.29)	
Stage III	70 (13.51)	27 (18.00)	22 (11.40)	21 (12.00)	
TBM diagnostic certainty, *n* (%)					<.001*
Confirmed	311 (60.04)	76 (50.67)	112 (58.03)	123 (70.29)	

Highly suspected	168 (32.43)	52 (34.67)	64 (33.16)	52 (29.71)	
Suspected	39 (7.53)	22 (14.67)	17 (8.81)	0 (0.00)	
GCS score, M (Q1, Q3)	14.00 (13.00, 15.00)	15.00 (12.00,15.00)	14.00 (13.00,15.00)	14.00 (13.00,15.00)	0.992
Drug Resistance Testing Performed, *n* (%)	81 (15.64)	13 (8.67)	38 (19.69)	30 (17.14)	0.016*
Drug-resistant TB, *n* (%)	16 (3.09)	5 (3.33)	11 (5.70)	0 (0.00)	0.007*
Imaging findings, *n* (%)					
Cerebral Infarction	213 (41.12)	46 (30.67)	74 (38.34)	93 (53.14)	<.001*
Tuberculoma	44 (8.49)	5 (3.33)	19 (9.84)	20 (11.43)	0.023*
Hydrocephalus, *n*(%)	136 (26.25)	34 (22.67)	42 (21.76)	60 (34.29)	0.012*
Intracranial Hemorrhage	8 (1.54)	1 (0.67)	5 (2.59)	2 (1.14)	0.401
Meningeal Enhancement	141 (27.22)	34 (22.67)	47 (24.35)	60 (34.29)	0.034*
LZD, *n*(%)	233 (44.98)	33 (22.00)	79 (40.93)	121 (69.14)	<.001*
FQs, *n*(%)	405 (78.19)	110 (73.33)	159 (82.38)	136 (77.71)	0.130
Amk, *n*(%)	111 (21.43)	34 (22.67)	50 (25.91)	27 (15.43)	0.046*
Mannitol, *n*(%)	424 (81.85)	132 (88.00)	164 (84.97)	128 (73.14)	<.001*
corticosteroids, *n*(%)	476 (91.89)	137 (91.33)	172 (89.12)	167 (95.43)	0.082
Prognosis, *n*(%)					0.201
Favorable Prognosis	395 (76.25)	122 (81.33)	145 (75.13)	128 (73.14)	
Unfavorable Prognosis	123 (23.75)	28 (18.67)	48 (24.87)	47 (26.86)	

* indicates statistical significance (P<0.05)

Supplementary Table S10 Sensitivity analysis of factors associated with unfavorable neurological outcomes after adjustment for admission period.

**Variables**	**β**	**S.E**	**Z**	**P**	**OR (95%CI)**
Admission year (2016-2019)	0.52	0.31	1.66	0.097	1.68 (0.91 ~ 3.10)
Admission year (2020-2023)	0.70	0.32	2.18	0.029*	2.02 (1.07 ~ 3.81)
Age >60	0.21	0.27	0.81	0.420	1.24 (0.74 ~ 2.09)
Hospital (Red Cross Hospital)	-0.89	0.28	-3.21	0.001*	0.41 (0.24 ~ 0.71)
Muscle Weakness	1.37	0.28	4.82	<.001*	3.94 (2.26 ~ 6.87)
MRC Disease Stage II	0.93	0.37	2.55	0.011*	2.54 (1.24 ~ 5.19)
MRC Disease Stage III	2.31	0.41	5.66	<.001*	10.11 (4.54 ~ 22.54)
White Blood Cell Count $>11\times10^9$/L	0.66	0.33	1.99	0.047*	1.93 (1.01 ~ 3.70)
Serum Albumin <30 g/L	0.91	0.33	2.78	0.005*	2.48 (1.31 ~ 4.71)
Hydrocephalus	0.64	0.26	2.49	0.013*	1.89 (1.14 ~ 3.11)

Note: OR, Odds Ratio; CI, Confidence Interval; S.E, Standard Error; β, Regression Coefficient. MRC, Medical Research Council;* indicates statistical significance (P<0.05).

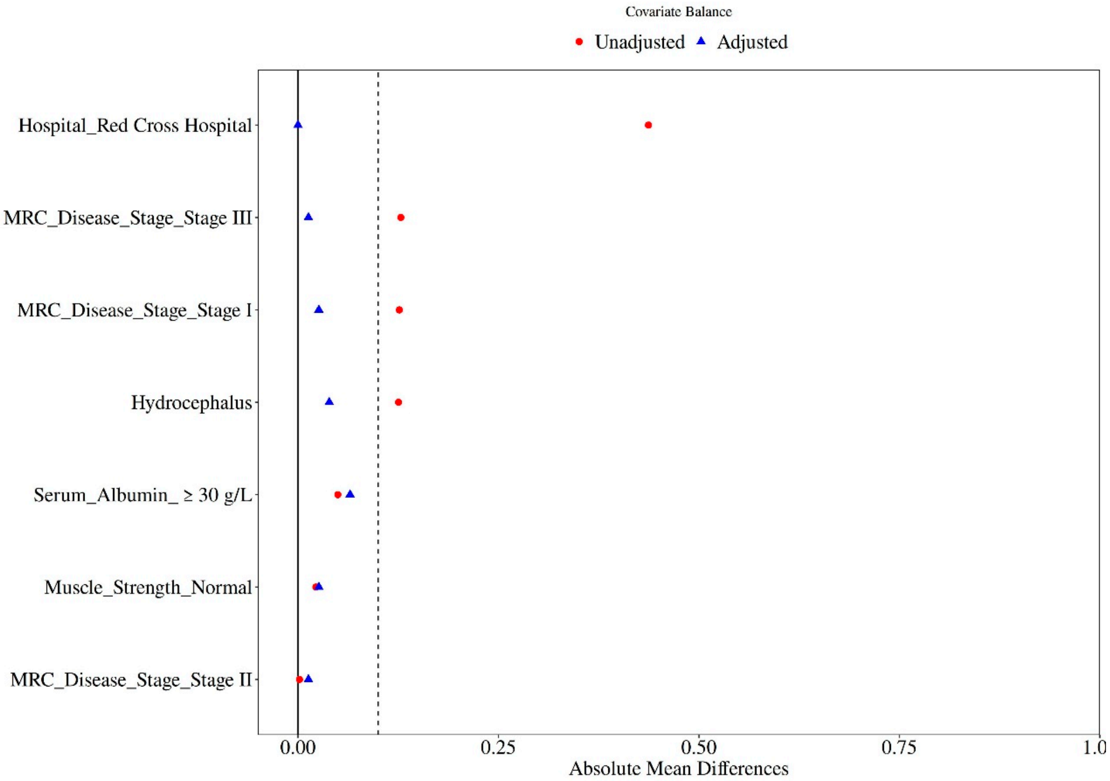

Supplementary Figure S1. Covariate balance before and after 1:1 propensity score matching according to intensified treatment exposure.

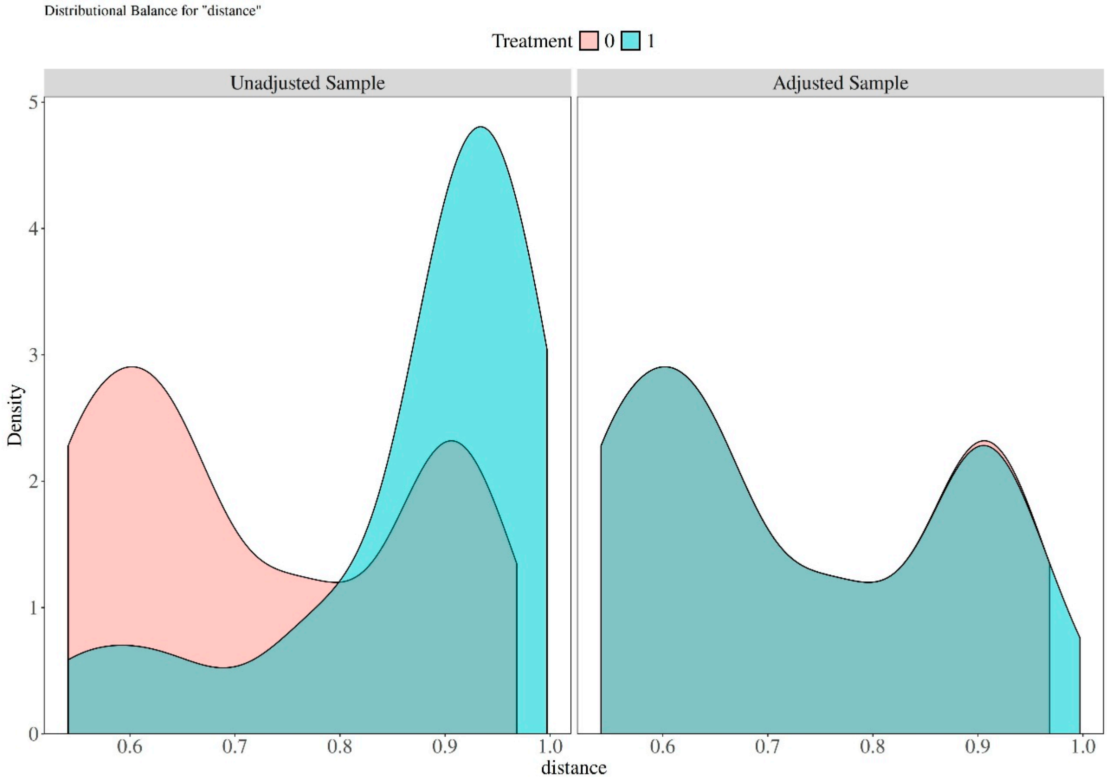

Supplementary Figure S2. Propensity score distribution before and after 1:1 nearest-neighbor matching to intensified treatment exposure.
